# Timely integration of palliative care into standard oncology care: An interview study with clinicians and patients with incurable cancer

**DOI:** 10.1017/S1478951525100333

**Published:** 2025-07-08

**Authors:** Carly S. Heipon, Natasja J. H. Raijmakers, Irene Dingemans, Anna K. L. Reyners, Yvette M. van der Linden, Linda Brom

**Affiliations:** 1 Department of Research & Development, Netherlands Comprehensive Cancer Organisation (IKNL), Utrecht, the Netherlands; 2 Department of Medical Oncology, University Medical Centre Groningen, University of Groningen, Groningen, the Netherlands; 3 Quality of Care, Dutch Federation of Cancer Patients Organisations, Utrecht, the Netherlands; 4 Centre of Expertise in Palliative Care, Groningen, University Medical Centre Groningen, University of Groningen, Groningen, the Netherlands; 5 Department of Radiotherapy, Leiden University Medical Centre, Leiden, the Netherlands; 6 Centre of Expertise in Palliative Care, Leiden University Medical Centre, Leiden, the Netherlands

**Keywords:** Timely palliative care, oncology, qualitative research, interview study, palliative care integration

## Abstract

**Background:**

Timely integration of palliative care (PC) into standard oncology hospital care offers significant benefits to patients with incurable cancer and their families. International recognition of the importance of timely PC has shifted the focus from integration to determining the optimal timing for introducing PC. The specific care responsibilities of oncology clinicians acting as generalists in PC and the optimal timing for involving PC specialists remain uncertain.

**Objectives:**

This study aimed to (1) explore how the concept of “timely PC” is understood by oncology clinicians and patients with incurable cancer and (2) investigate how PC is provided in a timely manner in daily clinical practice.

**Methods:**

An interview study was conducted with 18 oncology clinicians (7 physicians, 1 physician assistant, and 10 nurses/nurse practitioners) and 12 patients with incurable cancer. The interviews were conducted between October 2022 and June 2023 and a thematic analysis of the interviews was performed.

**Results:**

Three main themes emerged regarding “timely PC”: (1) timely PC is individual and situational, (2) identification of the right time is an ongoing challenge, and (3) proactive care is essential. Regarding the provision of timely PC, 3 themes were identified: (1) having a strong collaboration among various clinicians, (2) having the courage to start a clear and sincere conversation, and (3) being sensitive and personal.

**Significance of results:**

Being timely is not a fixed point in time, but depends on the individual patient and their situation. Clinicians should be proactive and gradual in bringing up PC-related topics and be careful to use the right words. Tools such as the surprise question can support in timely integrating PC but being timely PC highly depends on a patient’s individual context. Therefore, clinicians should be aware that timely PC is a constant search for the most fitting moment.

## Introduction

With the expected increase in cancer incidence over the coming decades, it has been predicted that by 2040 cancer will be one of the main drivers of palliative care (PC) needs (Bray et al. 2024; Etkind et al. 2017). Timely integration of PC into the care of patients with incurable cancer and their families has been shown to improve quality of life and satisfaction with care, reduce symptom burden and potentially inappropriate end-of-life care, and may even improve survival rates (Bakitas et al. 2015; Boddaert et al. 2020; Temel et al. 2010; Vanbutsele et al. 2018; Zimmermann et al. 2014). This extensive body of literature has led to international guidelines and recommendations from high-level associations, such as the American Society of Clinical Oncology (ASCO), which states that PC should be integrated within 8°weeks of diagnosis of advanced cancer (Ferrell et al. 2017). A consensus study among members of the Multinational Association of Supportive Care in Cancer (MASCC) and the European Society for Medical Oncology (ESMO) identified 13 indicators of oncology and PC (Hui et al., 2015a). This international recognition of the importance of timely PC has shifted the focus from *whether* PC should be integrated into standard oncology care to identifying the *optimal timing* for its introduction.

The Dutch healthcare system adopted an integrated generalist and specialist PC model, in which all clinicians provide basic PC based on their medical training. This includes basic symptom management and discussion of prognosis and treatment goals (Kaasa et al. 2018; Quill and Abernethy 2013). When needed, such as in complex symptom burden, clinicians in the hospital are supported by consultants of the specialist PC team (SPCT). These PC consultants are nurses, nurse specialists or physicians with additional expertise and broad experience in PC. Since 2017 every Dutch hospital caring for oncology patients is obliged to have an SPCT. Clinicians can call upon the PC consultants for peer-to-peer consultation or they can refer patients and their families. It has been suggested that the generalist-specialist model is the most feasible model for integrating PC into oncology, since there are not enough PC consultants to attend to all patients with PC needs (Periyakoil et al. 2022; Quill and Abernethy 2013). Ensuring timely PC within this model means both timely generalist PC: all clinicians should be able to identify potential PC needs and have adequate communication skills to discuss and manage basic PC needs, as well as timely specialist PC: clinicians should consider involving a PC consultant of the SPCT and do so when needed, in a timely manner.

Previous studies on the integration of PC in oncology have looked at when patients should be referred to a PC consultant, often using the word “early” instead of “timely.” “Early” was defined as a specific point in time (e.g. within 2 months of diagnosis of advanced cancer; Maltoni et al. 2016; Temel et al. 2017), prognosis (Zimmermann et al. 2014), or a combination (estimated prognosis of <12 months and within 3 months after diagnosis (Vanbutsele et al. 2018)). “Timely” PC does not necessarily refer to a specific point in time, but rather to care that is tailored to the needs of the patient and family, and provided at the optimal time and setting (Hui et al. 2022). However, there is no concrete definition of timely generalist PC that describes when oncology clinicians should integrate PC into their standard care. It also remains unclear which moment is the most optimal for timely referral to PC consultants (Hui et al. 2015a, 2018b, 2014).

Therefore, the aims of this study were (1) to explore how the concept of “timely PC” is understood by oncology clinicians and patients with incurable cancer and (2) investigate how PC is provided in a timely manner in daily clinical practice.

## Methods

### Study design

An interview study was conducted using semi-structured interviews. The Consolidated criteria for Reporting Qualitative research (COREQ) were used for reporting (Tong et al. 2007).

### Participants and setting

Three hospitals were selected based on the following information: prior participation in a Delphi study on integrated care, in which 21 Dutch hospitals took part (Heipon et al. 2024); data from a national survey of Dutch hospital-based SPCTs that included the assessment of PC integration indicators (e.g., inpatient services, outpatient clinics, interdisciplinary staffing, early referrals, symptom monitoring, and curriculum adherence) (Netherlands Comprehensive Cancer Center (IKNL) 2022); adherence to SONCOS guidelines, which serve as a benchmark for oncological care quality (Platform Oncology – SONCOS 2023); individual clinician interviews to further understand each hospital’s PC practices; hospital type (1 academic and 2 non-academic); and spread across the Netherlands. Based on these assessments, the research team reached a consensus on the final selection. To ensure a broad perspective on the timely integration of PC, different types of oncology clinicians (nurses, nurse specialists, physician assistants, and physicians) working in different departments of the hospital (medical oncology, hematology, gastroenterology and pulmonary) with and without additional PC training were invited to participate in an interview. Patients were recruited in the 3 participating hospitals. Eligible patients included inpatients and outpatients diagnosed with incurable cancer, aware of their diagnosis and able to speak Dutch. Patients were interviewed to obtain new insights and topics besides those obtained by the interviews with clinicians. Patients’ perspectives were considered complementary to those of clinicians.

### Recruitment

We used purposive sampling for the recruitment. The criteria for purposive sampling for clinicians were the following: treating patients with incurable cancer, having additional expertise and knowledge of PC, or not having this additional expertise. Clinicians were recruited via a designated contact in each hospital, who reached out to colleagues and forwarded the email addresses of those interested. The researcher then emailed them a description of the study and invited them for an interview. The interviews were conducted either at the hospital or online. Face-to-face participants received an informed consent form before the interview, and the online participants signed and returned the forms by email.

The criteria for purposive sampling for patients were the following: being diagnosed with incurable cancer and aware of this diagnosis, a minimal age of 18 years, and the ability to speak Dutch. Eligible inpatients were approached by their treating clinician or by the PC consultants of the SPCT and then contacted by the researcher (C.S.H.) to confirm that they had read the information and were willing to be interviewed. Eligible outpatients received the information leaflet during a visit to the outpatient clinic and, after consent, had their contact details securely emailed to the researcher (C.S.H.), who then arranged an interview at the hospital or the patient’s home.

### Data collection

Two topic lists were developed, 1 for clinicians and 1 for patients (Supplementary materials I and II). The topic lists were based on key publications regarding the timely integration of PC in oncology (Hui et al. 2015a; Kaasa et al. 2018) and the domains of PC as defined in the Netherlands Quality Framework for PC (Netherlands Comprehensive Cancer Center (IKNL) 2017). The patient topic list was pilot-tested with 2 patient representatives after which it was slightly adapted (Supplementary material II). All interviews were conducted between October 2022 and June 2023 by C.S.H., a female researcher with a background in anthropology. The average length of the interviews was 35 min for clinicians and 69 min for patients.

All interviews were audio-recorded and transcribed verbatim. The process of data collection was cyclical and iterative, with the researchers (C.S.H. and L.B.) analyzing the interviews after they had taken place and discussing emerging findings. Interviews were conducted until saturation was reached.

### Data analysis

Thematic analyses were conducted according to the 2 stages described by Braun and Clarke (2012) using the qualitative software package ATLAS.ti (version 23.1.1) (Muhr 1991). Interviews with clinicians and patients were analyzed separately. The analysis of the clinicians’ data was completed first. During the patient interviews and data analysis, many connections were observed. Therefore, it was decided to merge the 2 coding trees. Two researchers (C.S.H. and L.B.) independently coded 2 transcripts to establish the inter-observer reliability of the coding procedure. The constant comparative method was used to compare codes within and across interviews. C.S.H. coded the other transcripts and the codes were discussed in frequent meetings with the research team (C.S.H., L.B., and N.J.H.R.). Relevant quotations were selected to illustrate the themes.

### Ethical consideration

The study was conducted according to the Declaration of Helsinki. The Medical Ethics Committee of Brabant (NW2021-71) reviewed the study and exempted it from full approval of an ethics committee (CCMO, 2020). Written informed consent for participation (and recording) was obtained from all individual participants. In addition, the Dutch Personal Data Protection Act was followed in the data collection and analysis procedures.

## Results

A total of 18 clinicians and 12 patients were interviewed. The majority of clinicians were female (78%), and most patients were male (58%). The median age of the clinicians was 43 years (range 29–61), and the median age of the patients was 64 years (range 56–83). 72% of clinicians had additional training in PC (Table 1). In 7 patient interviews, the partner was also present during the interview to support the patient.

**Table 1.** Sociodemographic characteristics of clinicians (*n* = 18) and patients (*n* = 12)

	Clinicians	Patients
Age in years (median, range)	43 (29 − 61)	64 (56 − 83)
	*N* (%)	*N* (%)
Sex		
Female	14 (78)	5 (42)
Male	4 (22)	7 (58)
Hospital type		
Academic	6 (33)	5 (42)
Teaching hospital	5 (28)	2 (17)
Community hospital	7 (39)	5 (42)
Profession		
Oncology nurse	5 (28)	
Nurse practitioner oncology	5 (28)	
Medical oncologist	4 (22)	
Other physicians	4 (22)	
Additional training in PC	13 (72)	
Nine-day course in palliative care for physicians	5 (38)	
Two-year palliative care CME for physicians	1 (8)	
Nurses with 1-year palliative care CNE	7 (54)	
Marital status		
Married		7 (58)
In a relationship, living together		2 (17)
In a relationship, not living together		1 (8)
Living alone Widowed		2 (17)

CME, continuing medical education; CNE, continuing nursing education.

In exploring “timely PC,” 3 main themes emerged from the analysis of both the interviews of clinicians and patients, namely (1) timely PC is individual and situational, (2) identifying the right time is an ongoing challenge, and (3) proactive care is essential. In the context of providing timely PC in a generalist-specialist model, 3 further themes were identified, namely the importance of (1) having a strong collaboration among various clinicians (between both physicians and nurses, and PC-generalists and the PC consultants), (2) having the courage to start a clear and sincere conversation, and (3) being sensitive and personal (Table 2). All themes are described below from the perspective of clinicians and patients.

**Table 2.** Identified main themes per subject

1. Exploring the concept of “timely PC”	Identified main themes
	1.1 Timely PC is individual and situational
	1.2 Identifying the right time is an ongoing challenge
	1.3 Proactive care is essential
2. How timely PC is delivered a generalist-specialist model	Identified themes
	2.1 Having a strong collaboration between various clinicians
	2.2 Having the courage to start a clear and sincere conversation
	2.3 Being sensitive and personal

### Gaining a deeper understanding of timely PC

#### Theme 1.1: Timely PC is individual and situational

Clinicians found it challenging to define when initiating discussions about care preferences, possible future scenarios, or referring patients to a PC consultant was considered “timely.” Clinicians said it was difficult to identify a specific point in time that could be expressed in months or years. Rather, they spoke of moments in the disease trajectory, such as (shortly after) diagnosis of incurable cancer, when symptom burden is high, or when there is a possibility that the next treatment will not be effective.

I think it [timely] is when you get to a point where the therapy might cause problems or that you arrive at yet another line of treatment. But I find it quite difficult to be very black and white; when is timely? It depends on the individual, of what is going on, the context of the patient, and how urgent is it to have certain conversations with people. (Oncology nurse practitioner)

Clinicians reported that the timing varied from patient to patient and was strongly influenced by patients’ attitudes and coping strategies. Clinicians initiated advance care planning discussions earlier (e.g. shortly after diagnosis of incurable cancer) with patients who were open and had discussed their care wishes with family members compared to patients who were reluctant to discuss anything besides anticancer treatment.

More than half of our patients arrive with a stage IV disease, so that means that the treatment that is started is a palliative treatment. How much time and attention is spent on discussing the palliative part or the systemic therapy very much depends on the patient’s symptom burden and how the patient feels. If you have a very young person with a relatively low tumor load that you give a treatment that you most likely can prescribe for quite a long period, then discussions will be a bit more focused on symptoms that the patient copes with, the current treatment, and side effects. (Pulmonologist)

Clinicians did not identify a specific point in time with regard to the timely involvement of a PC consultant. Rather, they involved them there was a combination of aspects that made the situation difficult.

Often it’s the complex patients, for example, if they have a vulnerable situation at home which makes living at home impossible. Or patients with physical problems, such as pain that cannot be managed properly or in case of difficult palliative sedation. But also when you notice that a patient has a lot a lot going on for which you want additional expertise, then you often consult the specialist palliative care team. (Oncology nurse)

Clinicians who felt confident in providing generalist PC referred patients to a PC consultant when they felt they could not really reach the patient, could not put their finger on the patient’s problem, and therefore felt they could benefit from the fresh perspective of a PC consultant.

Especially if you have known a patient for a longer period of time you start to see him or her in a certain way. That’s when I like getting my colleagues’ opinion. And it’s also very nice for patients, who sometimes try very hard to tell me that everything is going great because I am the gatekeeper to their next treatment, to talk to someone else about what they would like if things are not going well. (Medical oncologist).

In their efforts to define timely PC, patients reflected on their own experiences and expressed that their perspective might be different from that of other patients. Having specific symptoms or needs (e.g. questions about their illness and prognosis or fears) and different moments in the disease trajectory (e.g. after being diagnosed with incurable cancer and being on the last line of anticancer treatment) were considered timely moments for integrating PC. Patients differed in whether they experienced feeling physically and mentally well as timely. Some did, while others wanted to focus on their anticancer treatment while things were going well.

I would have appreciated it if 2 weeks after [diagnosis of incurable cancer] there had been an appointment with a palliative care consultant who would have told me a bit more about what was going on. That my diagnosis does not mean that you die immediately and that you can still go through chemotherapy or radiotherapy, not to get better, but to prolong your life. (Patient)

#### Theme 1.2: Identifying the right time is an ongoing challenge

When trying to define timely PC, clinicians often referred to situations where PC was either integrated too late or too early. Examples of PC being integrated too late were in the case of a (medical) crisis or when patients were already experiencing a high symptom burden. However, discussing quality of life or introducing the possibility of meeting with a PC consultant at a patient’s first appointment after being diagnosed with incurable cancer was considered too early. At this time, patients may be too emotional and overwhelmed.

It’s just very difficult to make a proper assessment of how things are going to go. There are a lot of uncertainties. And patients can also become anxious if they are in an early phase of treatment, when you do not know if you are going to respond to a treatment, and you mention the specialist palliative care. It can make patients wonder if we are doing everything we can to make the treatment work. (Pulmonologist)

Clinicians also stated that they needed time to build trust and rapport before discussing possible future scenarios and a patient’s care wishes for when the anticancer treatment is no longer effective.

I do not do it [ask about care wishes or possible future scenarios] when I see people for the first time. Often, you have known patients a bit longer and therefore they feel a bit safer to talk about these topics. And some patients quite clearly say that they would like to leave it for now and that it will come in due course, while others give you a more extensive reply, that can be very different. (Oncology nurse practitioner)

Patients also felt that it was too early to start talking about PC during the appointment at which they were first told that their cancer was incurable. They expressed that they needed time before they felt able to discuss care preferences and treatment options. This time was needed to mentally process the diagnosis, the impact it might have on their lives, and to focus on the anticancer treatment.

In the first phase, the first 6 months, the first year, you need to get your thoughts in order and you have to start organizing things. During that time so much is happening in your life besides treatment. (Patient).

#### Theme 1.3: Being timely means being proactive

Clinicians’ examples of how they integrated PC showed a very proactive approach. They elaborated on looking ahead and actively discussing the possible consequences of certain treatment options, even when there was no immediate reason to do so or, for example, when patients had a life expectancy of months or years.

And what I also often try to do, when things are going well and you actually have time to spare, is not to think, well, things are going well now and I will be done in 10 min and I will be well ahead of my clinic, but to ask, well, how are things actually going? Or do you ever think about what it might be like in the future? (Oncology nurse practitioner)

According to clinicians, proactively integrating PC was a gradual process. This meant that PC topics were not discussed in a single conversation but were spread out over several appointments. Early in the disease trajectory, clinicians tend to focus on the physical dimension and explaining the disease. Later in the disease trajectory, they addressed topics related to other domains (psychological, social, and/or spiritual).

We know that if you talk about palliative care too late, people may feel that they have not had time to really think things through. So it is good to mention it early, but sometimes you have to take it step by step. For example, you might mention palliative care at a patients’ first appointment, just to give them something to think about. That also gives you a sense of whether the patient is open to it or not. (Pulmonologist)

Clinicians emphasized the importance of being able to prioritize what to discuss now and what to discuss later in order to integrate PC proactively and gradually. In their view, introducing a particular PC-related topic does not necessarily mean that it has to be fully explored. Topics might remain unaddressed for months, but briefly mentioning them early on can make it easier to discuss them in depth later.

Paying attention doesn’t mean that you have to do everything right away. But that you can say: “Well, I hear a number of things in your story that need attention (…). What are things that are important now and what can wait until next time? Or should we involve someone else to help with those worries?” You can also make a kind of roadmap with patients, what needs to be done today and what can be done another time. (Medical oncologist and consultant of the SPCT)

Patients varied in their willingness to proactively discuss non-physical issues, potential future scenarios, or their care preferences. Some wished they had been informed earlier about the possibility of seeing a PC consultant right after being diagnosed with incurable cancer, while others only felt ready when their symptoms worsened or when they reached the final stage of anticancer treatment. Patients also highlighted the importance of a gradual approach to integrating PC.

I do not think you need to talk about it every time. We have discussed it [end-of-life topics], so at this moment I do not see the need to discuss it again. By the time I get sicker we will pick it back up, that is how I feel. I do not think I need to talk about my final phase every time. I cannot say if that is different for someone else, but that is my opinion. I do not have to talk about my illness trajectory and how I am coping every time. (Patient)

### Providing timely PC in daily practice in a generalist-specialist model

#### Theme 2.1: Strong collaboration between clinicians

Clinicians explained that a strong collaboration was essential for the timely integration of PC. This included the collaboration between different disciplines (e.g. medical oncologists and spiritual counselors), between physicians and nurses, and between PC generalists and PC consultants. Clinicians stated that strong collaboration and short lines of communication allowed them to draw on each other’s expertise, providing different perspectives on patients’ symptoms. Additionally, clinicians highlighted how close collaboration between treating physicians and nurses contributed to earlier identification of PC needs, for they have different focuses and complementary skills in caring for patients. Nurses mentioned that their training, skills, and experience made them feel equipped to explore symptoms in all 4 dimensions: physical, social, psychological, and spiritual.

The advantage of being a nurse is that you see a patient very often. Compared to a doctor, you more often hear a patient say what they want or do not want. Another advantage is that a nurse does not have the ‘burden’ of medical knowledge, where you think: according to studies there is a 2% chance that a patient will make it through. Nurses just look at a patient and think about what it is they notice in a patient; is he deteriorating or not, last admission he was still walking, now he can no longer go to the toilet on his own? (Nurse turned physician assistant gastrointestinal and liver diseases)

Strong collaboration between PC generalists and PC consultants was seen as essential for timely integration of specialist PC. Clinicians mentioned that the approachability of PC consultants was key, not only in terms of logistics such as being easily accessible and having time to see patients, but more importantly through their open and supportive attitude. Consequently, clinicians did not experience any barriers to approach and involve a PC consultant.

They always respond very normally, it is never too much. They are never angry or irritated. When you call them, they always check if there is time. And they always say that they really appreciate you calling them and that they will make sure they come over. (Oncology nurse)

The role of nurses in the timely identification of PC needs was also highlighted from the patients’ perspective. Patients felt more comfortable expressing their feelings to nurses, because nurses dedicated time to listening, asked more and varied questions, and fostered a more personal connection.

The nurse asks about [my emotional well-being]. Last time when I spoke to her I cried and it all came out. I think the doctor is more for the treatment and with the nurse we have more conversations about my mental health and emotions. I don’t think the doctor has time for that, his responsibility and focus is more on the treatment I think. (…) [The nurse] asks different questions. It’s more of a chit-chat. With the oncologist you talk more about the kind of treatment we are going to do and with [the nurse] it is more about how things are going. Yes, more chit-chatting. Maybe more about the person behind the disease. (Patient)

#### Theme 2.2: Having the courage to start a clear and sincere conversation

Clinicians emphasized the importance of using the right words to integrate PC in a timely and well-received manner. This included providing clear explanations about PC, while being sincere about treatment prospects and potential side effects. According to clinicians, these conversations require courage, as the topics are sensitive and it is never certain how a patient will respond. Nevertheless, they emphasized the importance of having the courage to initiate these conversations.

When people are obviously incurably ill, I do not think you should hide that. So already at our first appointment I tell all my patients: you are not going to get better from this and the chances are very high that you will die from this. We still have these and these treatments to go, we are going to focus on that as well. We are obviously going to hope that you will live to be 100, but at the same time I also want to focus with you on what if you do not. (Medical oncologist)

Although they were sincere and clear, clinicians were still very careful about how they phrased things. They had seen how patients could become anxious or think that their health was rapidly deteriorating if PC-related topics were introduced without a certain degree of subtlety.

Over the years I have noticed how important it is how you deliver and introduce [PC-related topics]. Because people may be shocked and think: everything was going well and now you are bringing this up, that must mean that I will probably die soon. So you really have to introduce it in a sense of: it is not relevant right now because things are going really well, but it ever does become relevant, what do you think about this or that? Or do you already have wishes or thought about it or how do you feel about it? So that you bring it with some caution. (Oncology nurse practitioner and case manager)

Patients also reflected on the way in which PC was introduced during the course of their disease. They felt that the key was to give a clear explanation of what PC is and how it can support them and their families.

You have to start with telling people that there is a department and can help you to make it as bearable as possible, to ensure you keep enjoying the things you enjoy doing. And if you are missing anything, then they can see what you need. A special chair, a wheelchair, this or this or that. Then later you can say “and we call that palliative care.” (Patient)

When patients are first offered a consultation with the SCPT, they find it very important to be given a clear definition of PC and an explanation of the role of a PC consultant. A clear definition takes away any initial fears and associations with terminal care.

[When introducing the SPCT the physician assistant] said nowadays if you have cancer that does not mean you die immediately. There are so many good medicines with which people have a longer life expectancy. That is why it might be helpful to see the specialist palliative care team, so you can draw your horizon a bit more closer toward you. (Patient)

#### Theme 2.3: Being sensitive and personal

Clinicians were asked how they identified patients’ PC needs in a timely manner when they were not using a specific timeline to integrate PC. The examples they gave illustrated their tactfulness and sensitivity to issues that patients did not always express verbally. They explained that to achieve this, they used skills such as actively listening to patients’ stories and asking follow-up questions to uncover and identify the underlying PC needs.

Sometimes you do not measure being sick by blood values, you measure it by what someone will or will not be able to do in the future. So before anything else listen closely to the patient and do not rush into medical details and start a whole conversation based on medical reasoning. First hear how the patient sees their life and what they want or do not want to endure in order to undergo that treatment, because that is basically what it comes down to. (Nurse turned physician assistant gastrointestinal and liver diseases)

When patients were asked what they appreciated about their treating physician they often did not reflect on their technical skills or knowledge but instead focused on their attitude, using words such as involved, open, warm, compassionate, empathetic, gentle, humorous, sincere, and transparent. This attitude made patients feel comfortable asking anything and allowed for a more open discussion about treatment and care preferences.

You do notice that some doctors are more human-doctors, others are doctor-doctors. For example, whenever I call my own gastroenterologist she will call back the same day. When I called my urologist [previous treating physician] he did not call back. (…) He was very businesslike. As if I was just another patient. With all due respect, I did not feel like that was the doctor with whom I actually had a connection as a human being. I thought why do you not ask about my thoughts, what and how I feel, and what I choices I want to make? (Patient)

## Discussion

### Main findings

This qualitative study shows that clinicians and patients with incurable cancer view “timely PC” as individual and situational, rather than a fixed point in time. This requires a constant search for the most fitting moment to integrate PC, for which a proactive and gradual approach is essential. In daily clinical practice, PC is delivered in a timely manner through a strong collaboration between various clinicians. As clinicians find PC a sensitive topic they start integrating it by showing courage (just doing it), using the right words, and being sincere. Important communicative skills for discussing PC include being sensitive to issues underlying a patient’s story and maintaining a personal approach. Patients also value this personal connection, as it makes them feel they can ask and say anything.

### How communication and a clear understanding of PC can help in the search for the fitting moment

The finding that clinicians found it difficult to define timely PC in a concrete and unambiguous way is consistent with a qualitative study that found that clinicians had different understandings of when to initiate discussions about foregoing anticancer treatment at the end of life (Laryionava et al. 2015). That study found that clinicians use different approaches to initiate these discussions. The anticipatory approach, i.e. preparing patients gradually throughout the course of the disease, seems most in line with what both clinicians and patients in this study consider optimal for timely integration of PC. However, foregoing anticancer treatment is only one of many PC-related topics, moreover, at the end of life is late rather than timely. Other studies on timely integration of PC focus on timely referral to a PC consultant but do not discuss timely generalist PC (Crimmins et al. 2021; Hui et al. 2018b, 2022).

Our study shows that timely generalist PC is not one-size-fits-all and both clinicians and patients emphasized the importance of considering the individual patient and their situation. This ongoing assessment of a patient’s character and situation is particularly important as clinicians’ perspectives may differ from those of patients. For example, our study shows that when patients are feeling well, clinicians view this a timely moment to proactively integrate PC, whereas patients were more ambivalent about wanting to discuss PC when they were (still) feeling well.

Despite the difficulty of defining the concept of timely PC as a fixed point in time, some form of standardized moments at which to integrate PC is necessary. This standardization ensures that the timely integration of PC does not depend solely on clinicians’ training or patients’ preferences, thereby preventing PC from being integrated too late, as is often the case in current daily clinical practice (Adamidis et al. 2024; Boddaert et al. 2021). In the Dutch context of a generalist-specialist model, this standardization is 2-fold: standardized moments for integrating generalist PC (including symptom management and assessment and initiating advance care planning discussions) and standardized moments for involving a PC specialist. Standardization for both the integration of generalist and specialist PC can be embedded through care pathways (Groenewoud et al. 2021; van der Padt-Pruijsten et al. 2021), automated alerts in the electronic health record (Bush et al. 2018; Picker et al. 2017), or the development of guidelines and recommendations for the timely integration of PC into oncology (Ferrell et al. 2017; Hui et al. 2018a; Jordan et al. 2018; Kaasa et al. 2018; SONCOS, D.F.o.O.S 2017).

Results of this study show that the most optimal moment for integrating timely PC in a generalist-specialist model should be based on a patient’s needs rather than on a specific point in time (Hui et al. 2016). This calls for a structural discussion of patients’ needs and monitoring of symptoms in all 4 dimensions (physical, psychological, social, and spiritual). Studies have emphasized the importance of routine symptom monitoring, as it improves patient outcomes, including health-related quality of life and symptom control (Basch et al. 2017, 2016, 2022). While the literature regarding monitoring symptoms focuses mostly on finding the most fitting tools for both clinicians and patients, our study showed that most clinicians identify patients’ needs by picking up verbal and non-verbal cues. This emphasizes that identifying and monitoring symptoms is a communicative process, in addition to using a tool or interpreting patient-reported outcomes.

### The importance of clinicians’ communicative sensitivity and demeanor when providing timely PC

Our study shows that it is necessary to be clear and sincere in order to have timely discussions about PC in daily clinical practice. While this sounds straightforward, it is actually a difficult skill that requires communicative tact and subtlety. Other studies have also illustrated this difficulty by stating that clinicians should be honest but hopeful (Guetterman et al. 2024) or honest without being rude (Bensing et al. 2011).

Our findings show that timely PC integration requires sensitivity for picking up on patients’ non-verbal cues and for exploring issues expressed non-verbally, for example by asking follow-up questions. Another qualitative study showed that non-verbal cues were one of the ways in which GPs recognize that their patients are worried, and how picking up on these cues can effectively reassure patients (Giroldi et al. 2020). A grounded theory study stated that asking follow-up questions after verbal or non-verbal cues demonstrates empathetic listening, which makes patients feel heard and understood (Guetterman et al. 2024). While this communicative sensitivity may come more naturally to some clinicians than others, training and practice can improve active and empathic listening (Pehrson et al. 2016; Thangarasu et al. 2021).

Our study also found that patients appreciate a clinician who sees the person behind the disease and that patients were more likely to describe a clinician’s attitude than their technical skills when asked what they appreciated in their treating physician. This is in line with what another study calls “clinical demeanour,” which is explained as the subjective assessment of a clinician’s behavior and one of the clinical attributes through which patients experience empathy (Sanders et al. 2021). While clinical demeanor is difficult to capture, it is essential for being perceived as personal and empathetic. There are many communication trainings, some with a specific focus on cultivating empathy (Kelm et al. 2014), although their effect on patients’ outcomes remains unclear (Selman et al. 2017). A proposition paper from the European Society of Medical Oncology that elaborates on clinicians’ communicative tasks offers a possible explanation. It states that communication is more than a specific set of skills and requires constant judgment and interpretation of a patient’s situation and context. To improve clinicians’ competence and confidence, effective communication training should focus on clinicians’ lived experiences (their own feelings and attitudes) as well as society’s discourse about cancer.

### Strengths and limitations

A strength of this study is its inclusion of different perspectives, such as those of clinicians (both PC-generalist and PC-specialist), as well as those of patients, providing a comprehensive overview of the attitudes toward the timely integration of generalist and specialist PC. However, some limitations should be noted. First, clinicians were recruited through a contact person who was an oncology clinician, resulting in potential selection bias. To minimize bias, the researcher encouraged outreach to clinicians with less affinity with PC or who did not refer many patients to a PC consultant. Still, some PC-generalists expressed apprehension because they felt that they did not know enough about PC. Therefore, this may have led to the inclusion of PC-generalists with more expertise than the average generalist. Similarly, patient recruitment through treating clinicians or PC consultants may have introduced selection bias due to “gatekeeping,” in which clinicians may select certain patients and exclude others (Kars et al. 2016). To minimize the risk of bias, patients were recruited from different departments. Additionally, the varied perspectives, e.g. some patients preferring PC discussions soon after the diagnosis of incurable cancer, while others preferred focusing on treatment, were seen by the researchers as an indication of minimal bias. Another limitation is that patients can only reflect on their own disease trajectory, whereas clinicians can reflect on their practice based on a wide range of patients. Some patients found it difficult to identify the most appropriate time to discuss PC. We decided to merge the 2 coding trees to ensure a broad perspective on timely PC. Finally, the meaning of a term such as sincerity and what patients want to know about their illness and prognosis is largely influenced by a patient’s cultural background. This study predominantly included white Dutch patients who were aware of their diagnosis of incurable cancer. Thus, this study does not represent the cultural diversity of the Netherlands.

## Conclusion

Timely PC in the care for patients with incurable cancer is individual and situational and therefore is not a one-size-fits-all approach. Tools such as the surprise question can support in timely integrating PC but timely PC depends on a patient’s individual context. Therefore, finding the right time to integrate PC is a constant challenge and clinicians should be aware that timely PC is a constant search for the most fitting moment. It requires clinicians to frequently assess and interpret a patient’s situation and context. A proactive and gradual approach prevents clinicians from being too late and allows them to gauge how PC-related topics are received by the patient. Solid collaboration between different clinicians within the hospital allows for a more holistic view of patients and allows clinicians to easily call on each other’s expertise when needed. Patients appreciate tactful, sincere, and clear communication. These communicative tasks require clinicians to pick up on and respond to patients’ non-verbal cues and to reflect on their own visions and lived experiences.
